# Decoupling the Dynamics of Bacterial Taxonomy and Antibiotic Resistance Function in a Subtropical Urban Reservoir as Revealed by High-Frequency Sampling

**DOI:** 10.3389/fmicb.2019.01448

**Published:** 2019-07-02

**Authors:** Peiju Fang, Feng Peng, Xiaofei Gao, Peng Xiao, Jun Yang

**Affiliations:** ^1^Aquatic Ecohealth Group, Key Laboratory of Urban Environment and Health, Institute of Urban Environment, Chinese Academy of Sciences, Xiamen, China; ^2^University of Chinese Academy of Sciences, Beijing, China

**Keywords:** antibiotic resistance genes, bacterioplankton, microbial community, high-resolution temporal pattern, subtropical reservoir

## Abstract

Aquatic environments serve as important reservoirs of antibiotic resistance genes (ARGs), but the information on the high-resolution temporal pattern of ARGs in waterbodies is extremely limited. In this study, the weekly dynamics of ARGs and their relationships with microbial taxonomic communities and environmental variables were analyzed in a subtropical urban reservoir over the period of 1 year using high-throughput approaches. In total, 197 ARGs and 10 mobile genetic elements (MGEs) were detected. The results showed that the bacterial community had a seasonal pattern, while ARGs composition did not exhibit seasonality, thereby indicating the asynchrony or decoupling of temporal patterns of microbial taxonomy and function. More importantly, bacterial abundance and community diversity were more strongly correlated with 17 measured environmental variables than ARGs (36 significant correlations for OTUs, 11 for ARGs). However, stochastic processes appeared to have a minor role in the structuring of the ARG profiles, but a more important role in the structuring of bacterial taxonomic communities. Furthermore, we found that precipitation and turbidity were significantly correlated with the richness and diversity of ARGs, suggesting that multiple environmental factors influence the composition and dynamics of ARGs in complex ways. MGEs were abundant and showed significant positive correlations with ARGs, indicating a plausible influence of MGEs on the variation of ARGs. This is the first study which provides an overview of high-resolution dynamics of ARGs in a subtropical waterbody. Our results improve the understanding of microbial processes and mechanisms of ARGs at fine temporal scale, and offer empirical data of use in the monitoring, assessment and management of the urban water environments.

## Introduction

In aquatic ecosystems many studies have been carried out on nitrogen, phosphorus, and microbial community (Yang et al., 2012; Zhang et al., 2019), however various “emerging” pollutants including antibiotics and antibiotic resistance genes (ARGs) have been rapidly drawing increased attention from the public and government (Pruden et al., 2006; Zhu et al., 2017; Yang et al., 2018). Antibiotics, first discovered by Sir Alexander Fleming in 1928, were regarded as panacea for any microbial infections (World Health Organization [WHO], 2014). However, with the emerging problem of antibiotic resistance, antibiotic resistant bacteria (ARB) and ARGs have been identified as a significant issue, posing a serious threat to the health of humans and the environment (Pruden et al., 2006; Berendonk et al., 2015). Antibiotic resistance is ancient (D’Costa et al., 2011), but with the continuous increase in discharge of overused antibiotics, the development, spread, and enrichment of ARGs has accelerated globally (Laxminarayan, 2014), and heavy metal has been reported to play an important role in the enrichment of antibiotic resistance by co-selection mechanisms (Stepanauskas et al., 2006). More importantly, ARGs can be exchanged and transferred among environmental bacteria, pathogens and non-pathogens via horizontal gene transfer (HGT) carried out by mobile genetic elements (MGEs) (Kruse and Sørum, 1994; Martínez et al., 2015). Aquatic environments are considered to be significant reservoirs of both ARB and ARGs, providing ideal settings for the occurrence and dissemination of ARGs (Marti et al., 2014). Due to long-term misuse and overuse of antibiotics by human, urban water often contains diverse ARGs at high levels (Xu et al., 2016), which may pose high risk to human health during the interaction with receivers. Therefore, ARGs occurrence and their temporal dynamics in the aquatic environment have become an important issue in environmental science (Yang et al., 2018).

Microbial communities can vary between environments, and play an important role in drive global biogeochemical cycling, but it is not well known how variation in taxonomic composition relates to function (Louca et al., 2016). Previous studies reported that bacterial community composition closely correlates with ARG profiles and controls the transfer of ARGs in antibiotic-rich environments (e.g., soil, urban wastewater, and sewage sludge) (Forsberg et al., 2014; Su et al., 2015), but the contribution of microbial community shifts to antibiotic resistance function variations in natural waterbodies with low and medium antibiotic concentrations remains largely unknown, especially at a fine temporal scale. Furthermore, microbial communities have been reported to exhibit seasonal variability in the environment (Treusch et al., 2009), and high-frequency sampling analysis could reveal and disentangle detailed temporal dynamics of bacterial community (Lindh et al., 2015). Previous studies have assessed the temporal variation of ARGs and supported the occurrence of seasonal variations of ARGs based on only two or four sampling time points in 1 year (An et al., 2018; Zheng et al., 2018). However, research on the dynamics of ARGs in urban water environment, and their relationship to microbial taxonomic community and environmental factors based on high-frequency time series is still lacking.

Revealing the mechanisms that drive the variation of microbial communities and ARG profiles are major challenges. It has been shown that microbial communities are simultaneously shaped by stochastic (neutral) and deterministic processes (Sloan et al., 2006; Logares et al., 2013). The neutral model is a stochastic process that develops community through births, death and immigration, can successfully predict microbial community assembly dynamics (Hubbell, 2001). However, the neutral model cannot completely descript the community assembly without deterministic process such as environmental factors and competition (Sloan et al., 2006; Ofiţeru et al., 2010). Both stochastic (neutral) and deterministic processes need to be considered when investigating the assembly of microbial communities and ARG profiles, but there is still a lack of studies on the contribution of both processes in structuring ARG profiles (Zhu et al., 2017; Guo et al., 2018).

The urban reservoir in this study is located in a rapidly urbanizing area of Xiamen, it receives numerous pollutants from surrounding housing estates by point and non-point source pollutions. However, this urban reservoir is the most important landscape waterbody in the Jimei district, so the ARB and ARGs in the water may pose ecological risk in the interaction with inhabitants and tourists. In this study, we simultaneously investigated the dynamics of bacterial OTUs and ARGs in the urban reservoir using high-throughput qPCR and 16S rRNA gene sequencing on a weekly basis over a 1-year period. We aimed to (1) characterize the temporal dynamics of both bacterial taxonomic community and antibiotic resistance gene profiles; (2) reveal the relationships between bacterial taxonomic composition and antibiotic resistance function at different levels of taxonomic resolution; and (3) explore the community assembly processes and mechanisms shaping the ARGs composition dynamics. Additionally, we hypothesize that the temporal patterns of microbial OTUs and ARGs are asynchronous, and their temporal patterns are influenced by different processes and mechanisms. To our best knowledge, this is the first study to analyze a high-resolution time series of bacterial OTUs and ARGs communities in urban water environments. Therefore, this study may provide a fundamental data for evaluating the ecological risks of ARGs in urban water environment and offer useful information for government and policy maker.

## Materials and Methods

### Study Area and Sample Collection

All samples were collected once a week from September 2016 to August 2017 in station G (24°36′ N, 118°04′ E) of Xinglinwan Reservoir, which is situated in the Houxi River watershed, Xiamen city, Fujian province, southeast China (Supplementary Figure S1). In total, 51 samples were collected in this study. Several stormwater and wastewater inlets were found near the station G along the reservoir shoreline within a distance of 1000 m. The Xinglinwan Reservoir is a subtropical eutrophic urban reservoir, and is characterized by high turbidity and low water transparency. It has multiple functions in flood control, aquaculture and tourism, exerting a great impact on the surrounding residents.

Surface waters were collected in a sterile container from the top 0.5 m water, immediately transported to laboratory and kept in the dark until pre-treatment within 0.5 h. In order to remove the influence of large plankton and particles, samples were pre-filtered through a 200 μm mesh, and then filtered through 0.22 μm polycarbonate membranes (47 mm diameter, Millipore, Billerica, MA, United States) using a vacuum filtration system. To ensure the sufficient microorganisms, the filtrated water volume ranged from 200 to 500 mL for all samples, because we kept a similar filtrating time (about 50 min) for each of membranes. The membranes were placed in sterilized tubes and stored at –80°C until DNA extraction.

### Environmental Parameters

A total of 17 environmental variables were determined in this study (Supplementary Figure S2). The comprehensive trophic state index (TSIc) were calculated according to Yang et al. (2012). Water temperature (WT), pH, dissolved oxygen (DO), turbidity, electrical conductivity (EC), salinity, and oxidation reduction potential (ORP) were measured *in situ* with a Hydrolab DS5 multiparameter water quality analyzer (Hach Company, Loveland, CO, United States). Chlorophyll *a* concentrations were quantified by PHYTO-PAM Phytoplankton Analyzer (Heinz Walz GmbH, Eichenring, Germany). Total carbon (TC), total organic carbon (TOC), total nitrogen (TN), ammonium nitrogen (NH_4_-N), nitrate nitrogen (NO_3_-N), nitrite nitrogen (NO_2_-N), total phosphorus (TP), and phosphate phosphorus (PO_4_-P) were measured according to the standard methods described in our previous study (Liu et al., 2013). The precipitation data referred to the cumulative rainfall during the last week before the sampling day, and the data were collected from the Xiamen Meteorological Bureau.

### DNA Extraction and High-Throughput Sequencing

Total DNA was directly extracted from each membrane using the FastDNA SPIN Kit (MP Biomedical, Santa Ana, CA, United States) according to the manufacturer’s protocol, and subsequently the concentration and quality of the DNA were determined using a NanoDrop 1000 spectrophotometer (Thermo Fisher Scientific, Waltham, MA, United States).

To investigate the bacterial taxonomic community, the V3-V4 hypervariable regions of the 16S rRNA gene were amplified, purified, and quantified according to our previous procedure (Guo et al., 2018). The PCR products were pooled and sequenced on the Illumina Miseq platform (Illumina, Inc., San Diego, CA, United States) using a paired-end (2 × 250 bp) sequencing strategy. All the raw sequences were quality-controlled using MOTHUR v1.39.0 (Schloss et al., 2009). The unoise3 pipeline was used to pick operational taxonomy units (OTUs) at 3% dissimilarity level (Edgar, 2010). OTU sequences were taxonomically classified by USEARCH (sintax) against the Greengenes database (DeSantis et al., 2006). All eukaryotic, chloroplast, archaeal, mitochondrial and unknown sequences were removed from the data set. Finally, all sequences were normalized to 50,014 sequences for each of 51 samples, and these sequences were clustered into 9834 OTUs.

The raw data for 16S rRNA gene analysis in this study have been deposited in the NCBI sequence read archive (SRA) database under the BioProject number PRJNA510463 and the accession number SRP173857.

### High-Throughput Quantitative PCR (HT-qPCR)

High-throughput quantitative PCR was performed to detect the abundance of ARGs using the Wafergen SmartChip real-time qPCR platform (Wafergen Biosystems, Fremont, CA, United States). A total of 296 primer sets targeting 285 resistance genes, 8 transposase genes, the class I integron-integrase gene (*intI*), the clinical class 1 integron-integrase gene (*cIntI*), and 16S rRNA gene were used. These primer sets and PCR reaction protocols were listed in our previous studies (Guo et al., 2018; Liu et al., 2018). Three technical replicates were performed for each sample, and non-template negative control was also included for each primer set. The absolute abundance of ARGs and MGEs was calculated by normalizing their abundance to the absolute 16S rRNA gene copy number, which was estimated by real-time quantitative PCR (Ouyang et al., 2015) as described below.

### Real-Time Quantitative PCR (qPCR)

Real-time quantitative PCR was used to quantify the absolute copy number of 16S rRNA genes in all samples. It was performed on a Lightcycler 480 instrument (Roche, Basel, Switzerland). The 20 μL reaction contained 10 μL 2× LightCycler 480 SYBR Green I Master Mix (Roche, Basel, Switzerland), 7 μL nuclease-free PCR-grade water, 2 μL diluted DNA, and 0.5 μM of each primer. The reactions were performed in triplicate with negative controls. The following thermal cycling conditions were used: initial incubation at 95°C for 5 min, followed by 40 cycles of 95°C for 15 s, 60°C for 1 min, and 72°C for 15 s.

A six-point calibration curve was generated from 10-fold dilutions for an external standard calculation. The melting curves were used to analyze the specificity of PCR products, and PCR efficiency ranged from 96 to 105% in this study.

### Statistics

The Bray-Curtis similarity was calculated using PRIMER v7.0 (Clarke and Gorley, 2015), and non-linear regression of time lag with community similarity was analyzed to reveal the temporal patterns of bacterial OTUs and ARGs communities. The non-linear regression was the fit to the sinusoidal curve to explore the seasonality in SigmaPlot v12.0 (Systat Software Inc., Chicago, IL, United States). The diversity indexes were calculated using the vegan package in R v3.3.1. The SIMPER analysis was performed to identify the contribution of bacterial OTUs and ARGs to the temporal variation of communities or profiles (Clarke and Gorley, 2015).

Spearman’s and Pearson’s correlations were calculated using SPSS v20.0 (IBM Corp, Armonk, NY, United States), and the Mantel test was performed in an R environment with the vegan package. Further, a Procrustes test was conducted with the vegan package in R environment to explore the synchronicity of bacterial OTUs communities and ARG profiles (Forsberg et al., 2014). Network analysis was used to reveal Spearman correlations between ARGs and bacterial taxa with the picante package in R v3.3.1, and the correlation coefficients |*ρ*| *>* 0.6 between microbial taxa and ARGs, and |*ρ*| *>* 0.4 between MGEs and ARGs, were considered at *P* < 0.01. Network information was generated by the Gephi v8.2 and Cytoscape v3.6.1.

In addition, we used redundancy analysis (RDA) to explore the relationship between environmental factors and the compositions of bacterial OTUs and ARGs subtypes. We further used partial redundancy analysis (pRDA) to evaluate the relative contribution of bacterial taxonomic community, environment variables and MGEs to the ARG profiles of all samples by R v3.3.1 with the vegan package. To reveal the assembly processes underlying both bacterial OTUs and ARGs compositions, we used the neutral community model, which can evaluate the importance of stochastic processes in explaining the assembly of bacterial taxonomic and functional communities (Sloan et al., 2006). This model predicts the relationship between the frequency at which taxa occur in a set of local communities (in this case, bacterial OTUs and ARGs at one sampling point) and their abundance across the wider metacommunity (bacterial OTUs and ARGs sampled over a year). The neutral models were performed in R v3.3.1 using the Hmisc, stats4, and minpack.lm packages.

## Results

### Dynamics of Environmental Variables, Bacterial OTUs, and ARGs Compositions

The major physicochemical variables exhibited a great change over time (Supplementary Figure S3). Comprehensive TSIc varied from middle eutrophic level to hypereutrophic level, and nutrient concentrations were high throughout the study period, with higher value in 2017 than 2016. Water temperature ranged from 15 to 35°C during the 1-year sampling. Higher precipitation values were recorded in September 2016 and June 2017.

We found 197 unique ARGs and 10 MGEs in this study, and the ARGs covered almost all major classes of antibiotics commonly used. The number of ARGs detected in each sample varied from 18 to 74, representing three major resistance mechanisms including antibiotic deactivation (53.8%), cellular protection (28.6%), and efflux pump (16.9%) (Supplementary Figure S4). The absolute abundance of ARGs ranged from 4.03 × 10^6^ copies/L to 3.72 × 10^9^ copies/L (mean ± s.e. = 4.55 × 10^8^ ± 1.11 × 10^8^), while the absolute abundance of MGEs varied from 6.62 × 10^5^ copies/L to 1.84 × 10^9^ copies/L (1.92 × 10^8^ ± 5.28 × 10^7^). The Shannon-Wiener index of bacteria ranged from 4.95 to 7.58 (Supplementary Figure S5). The bacterial community was dominated by Actinobacteria, Proteobacteria and Cyanobacteria, while Proteobacteria was the most abundant phylum in September and October (36.1% ± 2.5%, *n* = 8), and Actinobacteria dominated the bacterial community most of the time (37.9% ± 1.7%, *n* = 43).

**Figure 1:**
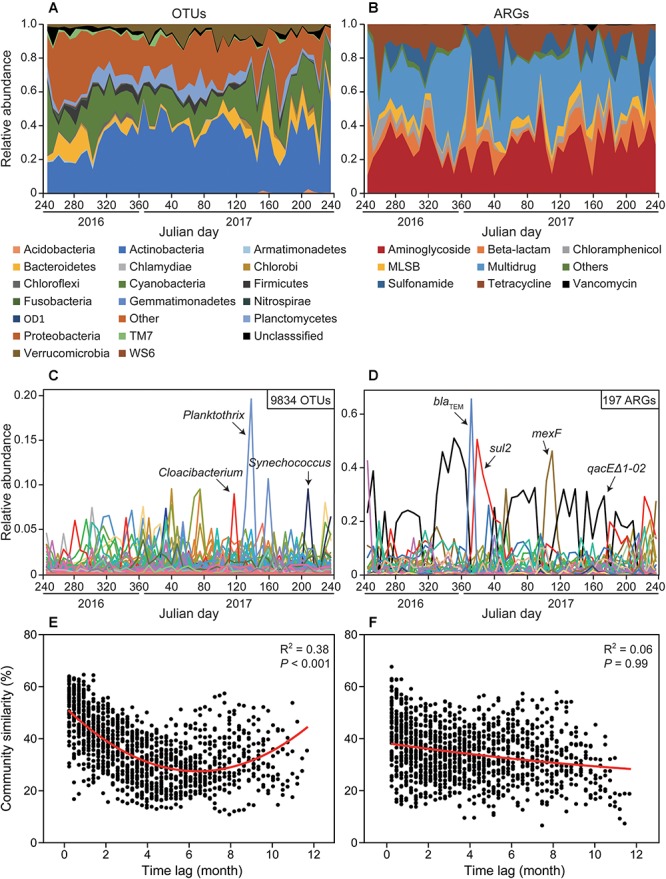
Contrasting dynamics of bacterioplankton OTUs and antibiotic resistance genes (ARGs) from September 2016 to August 2017 in Xinglinwan Reservoir. Although relative abundances of bacteria at phylum level **(A)** and ARG at type level **(B)** appeared to be relatively stable, the relative abundances of bacterioplankton OTUs **(C)** and ARG subtypes **(D)** varied extensively and rapidly. Time-lag regression analysis showing temporal dynamics of bacterial community **(E)** and ARG profiles **(F)**. The red lines represent non-linear regression model fit to month lag versus Bray-Curtis similarity (%), and only bacterial community results in a good fit of a sinusoidal curve, indicating only bacterial community has the evidence of seasonality.

**Figure 2:**
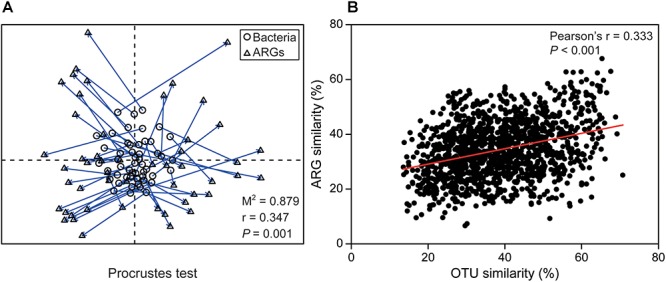
The relationships between bacterial community and ARG profiles. **(A)** Procrustes test of the significant correlation between ARG composition and bacterial community composition based on Bray-Curtis similarity metrics (*M*^2^ = 0.879, *r* = 0.347, *P* = 0.001, 999 permutations). **(B)** The significant but weak correlation between bacterial taxonomic community similarity and ARG profiles similarity.

Time series analysis indicated relative stability at bacterial phylum and ARG type level, but very rapid or abrupt fluctuations among both bacterial OTUs and ARG subtypes (Figure 1). The relative abundance of ARG subtypes exhibited distinct variations across the year at weekly intervals (Figure 1D). For example, the relative abundance of *qacE*Δ*1-02* (multidrug resistance gene) varied from 0 to 0.5 and its relative abundance changed almost every week. Time-lag regression analysis showed that the bacterial community displayed a good fit to a sinusoidal curve (Figure 1E), but ARG profiles failed to fit such a curve (Figure 1F); indicating that bacterial community composition exhibited seasonality while ARGs composition did not. The general variation of ARG similarity was narrower compared to the bacterial taxonomic community (Supplementary Figure S6), suggesting that the compositions of resistomes were more stable. Additionally, SIMPER analysis indicated that Proteobacteria contributed most to the variation within the microbial community (31.86%), while beta-lactamase resistance gene contributed most to the variation of ARGs (23.41%) (Supplementary Table S1). Furthermore, Procrustes test revealed asynchrony or decoupling between bacterial ARGs and OTUs communities (*r* = 0.347, *P* < 0.01, Figure 2A). However, the Pearson’s correlation indicated a significant but weak relationship between bacterial ARGs and OTUs communities (Figure 2B).

### Co-occurrence Patterns Between Bacterial Taxonomic Community and Antibiotic Resistome

**Figure 3:**
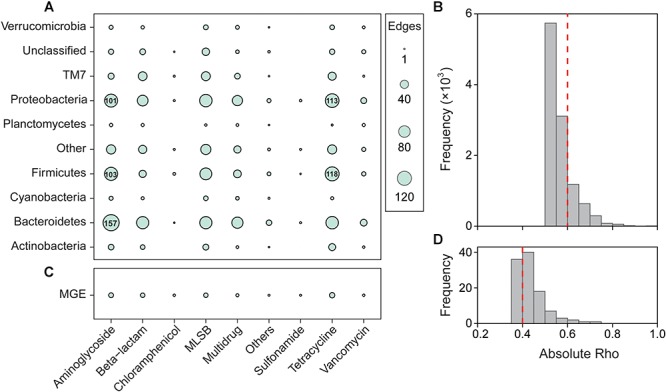
Significant correlation among bacterial OTUs, ARGs, and MGEs. Classification of nodes within the network depicting the co-occurrence patterns of microbial taxa and ARGs **(A)**, MGEs and ARGs **(C)**. Other, other bacterial phyla; others, other ARGs. The Spearman’s absolute rho was calculated at OTU and ARG subtype levels, and the number of edges was summarized at phylum and type levels, respectively. The edges correspond to a strong (Spearman’s |ρ| > 0.6 for microbial taxa and ARGs, Spearman’s |ρ| > 0.4 for MGEs and ARGs) and significant (*P* < 0.01) correlation between nodes. Frequency distributions of correlation coefficients between ARGs and microbial taxa **(B)** and between ARGs and MGEs **(D)** at *P* < 0.01.

Network analysis was performed to reveal the significant and robust correlations between ARG subtypes and bacterial OTUs. The whole network consisted of 1,547 nodes and 2,139 edges, and showed a modularity index of 0.762. Aminoglycoside resistance genes had the most edges (489) with bacterial taxa, followed by tetracycline (474), MLSB (454), and beta-lactamase (318) resistance genes (Figure 3A,B). Meanwhile, Proteobacteria, Bacteroidetes, Firmicutes, and TM7 were significantly correlated with most ARGs. The co-occurrence network showed that the hubs of all modules were ARGs (Supplementary Figure S7A), indicating the important roles of ARGs in the network.

We also used Mantel tests to evaluate the relationships between bacterial phyla and ARG types. Similar to the results of the network analysis, most ARGs were significantly and strongly correlated with Bacteroidetes, Firmicutes, and Proteobacteria (Supplementary Table S2). However, sulfonamide resistance genes did not show any significant correlation with the bacterial community, and MGEs only significantly correlated with Chloroflexi, Firmicutes, and TM7.

### Relationships Between Environmental Factors, Bacterial OTUs, and ARGs

**Figure 4:**
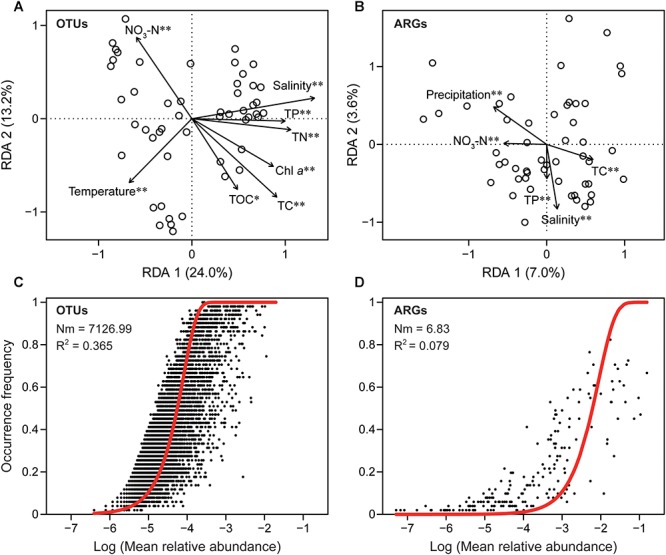
The different assembly mechanisms of bacterial community (taxonomy) and ARG profiles (function). Redundancy analysis (RDA) showing the significant relationship between environmental factors and bacterial community **(A)**, environmental factors and ARGs **(B)**. Chl *a*, chlorophyll *a*; TC, total carbon; TOC, total organic carbon; TN, total nitrogen; NO_3_-N, nitrate nitrogen; TP, total phosphorus. Only factors with significant correlation were included. ^∗^*P* < 0.05; ^∗∗^*P* < 0.01. Fit of neutral model for bacterial taxonomic **(C)** and ARG functional **(D)** communities. Red lines represent the best fit to the neutral model. Nm indicates metacommunity size times immigration, *R*^2^ indicates the fit to the neutral model.

Mantel tests showed aminoglycoside, beta-lactamase, MLSB, tetracycline, and vancomycin resistance genes were significantly correlated with precipitation (Supplementary Table S3). RDA further indicated that water temperature, chlorophyll *a*, salinity, total carbon, TOC, total nitrogen, nitrate nitrogen, and total phosphorus were significantly correlated with the β-diversity of the bacterial community, while salinity, total carbon, nitrate nitrogen, total phosphorus, and precipitation were strongly correlated with ARGs compositions, suggesting that nutrients were key factors that influence the dynamics of both ARGs and bacterial communities (Figure 4). We found that the bacterial community (*R*^2^ = 0.365) showed a much better fitted to the neutral model (Figure 4) than ARGs (*R*^2^ = 0.079), indicating that stochastic process played an important role in microbial community assembly but not in the resistome profiles.

**Figure 5:**
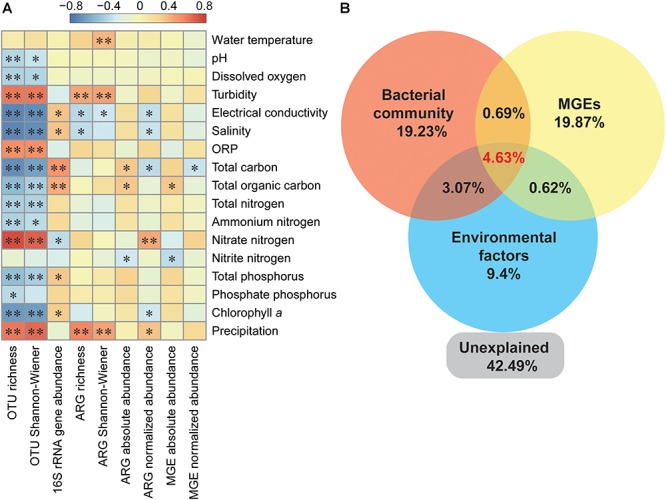
Variation of ARG profiles explained by different ecological variables. **(A)** Spearman’s correlation between bacterial OTUs, ARGs, MGEs, and environmental parameters. ORP, oxidation reduction potential. ^∗^*P* < 0.05; ^∗∗^*P* < 0.01. **(B)** Partial redundancy analysis illustrating the effects of bacterial community (relative abundance of ten major phyla), MGEs and environment factors on ARGs distribution pattern. Environmental factors referred to significant correlated factors, including salinity, total carbon, nitrate nitrogen, total phosphorus, and precipitation.

The absolute abundance of ARGs was positively correlated with total carbon and TOC, while ARGs richness was significantly and positively correlated with turbidity and precipitation (Figure 5A). Additionally, turbidity and precipitation also exhibited a positive correlation with richness and α-diversity of bacterial community, but were not significantly correlated with 16S rRNA gene abundance (Figure 5A). Partial redundancy analysis showed that the bacterial community, MGEs and environmental factors separately explained 19.23, 19.87, and 9.4% of the variation of ARG profiles, respectively. Interaction between all variables explained 4.63% of the variation, and 42.49% of the variation was unexplained.

### Correlations Between ARGs and MGEs

The absolute abundance of ARGs was positively correlated with MGEs (Supplementary Figure S8), and the normalized abundance of MGEs was significantly correlated with the richness and normalized abundance of ARGs (Supplementary Table S4). The network analysis further indicated that aminoglycoside, beta-lactamase, MLSB, and tetracycline resistance genes were significantly correlated with MGEs (Figure 3C,D), and the co-occurrence pattern revealed that *Tp614* and *tnpA-03* play the most important roles in the network (Supplementary Figure S7B). However, the integrase gene *intI-1* only showed a significant correlation with multidrug and vancomycin resistance genes (Supplementary Table S5), but the absolute abundance of transpose gene *tnpA-05* showed a strong correlation to all ARG types except vancomycin resistance genes (Supplementary Table S6). Additionally, the transposase genes *IS613*, *tnpA-03*, *tnpA-04*, and *tnpA-07* also showed a significant correlation with some types of ARGs. Partial redundancy analysis showed that 19.87% of the variation of ARG profiles was explained by MGEs (Figure 5B), indicating that MGEs were an important contributor to the change of ARG profiles during our study period. These results suggested that MGEs played a crucial role in the change, accumulation and dissemination of ARGs in this urban landscape reservoir.

## Discussion

### The Contrasting Temporal Patterns Between Bacterial OTUs and ARGs

Our high-frequency time series-based study revealed clear differences in the temporal patterns of microbial communities and ARG profiles, along with the decoupling of the temporal dynamics of bacterial taxonomy and antibiotic resistance function in an urban waterbody. The composition of ARG subtypes varied frequently over the year but was relatively stable at the ARG type level. None of the ARG subtypes was present in all 51 samples, and no distinct seasonal patterns in ARG profiles could be determined, but microbial taxonomic community had significant seasonal pattern.

ARGs in this urban reservoir were detected at relatively high abundance and richness. The diversity of ARGs in this study was greater than that reported from Chinese suburban/rural lakes/reservoirs (Liu et al., 2018). The high abundance and richness in this waterbody may be driven by the combined human activities of sewage discharge and agricultural/urban runoff, as it is generally known that these activities increase the number of ARGs in waterbodies (Zhu et al., 2017; Ahmed et al., 2018; Liu et al., 2018). Moreover, aminoglycoside and multidrug resistance genes dominated the abundance of ARGs in this study, while aminoglycoside and beta-lactamase resistance genes dominated the types or richness of ARGs. In contrast, Jia et al. (2017) found in swine wastewater and downstream water that tetracycline and aminoglycoside resistance genes were the dominant ARGs, perhaps due to the different antibiotic selection pressures in different environments. However, the primer limitation of this study may result in the bias of detected ARGs, as the PCR-based method only covered limited number of ARG types. Additionally, a majority of ARGs investigations have been carried out in municipal wastewater treatment plants. An et al. (2018) showed significant changes of ARG profiles based on samples just collected in February and August, while another study based on monthly samples for 1 year did not find obvious seasonal variation in methicillin-resistant gene although it varied between months and sampling sites (Börjesson et al., 2009). Yang et al. (2013) suggested that some types of ARGs in activated sludge showed seasonal fluctuations during a 4-year investigation, while most types of ARGs did not show seasonal fluctuations, but these results were based on samples collected only in summer and winter. However, according to our study, ARG subtypes were extremely dynamic and some of them frequently showed rises and falls over short-time periods (i.e., in just one week) but did not show seasonal pattern, so the previous low-frequency based studies may not completely and effectively reflect actual seasonal dynamic patterns of ARGs at fine temporal scale. Overall, our study based on high-frequency sampling over long timescales provide better understanding of the detailed temporal pattern of ARG profiles.

Our data revealed that some bacterial OTUs (e.g., *Planktothrix*) fluctuated rapidly, while major phyla remained stable at a broad taxonomic level. The microbial community showed an obviously seasonal variation and a trend of resilience during 1 year (12 months). In the microbial community, Proteobacteria and Actinobacteria were the dominant phyla, consistent with previous studies in drinking water system (Jia et al., 2015) and rivers (Jia et al., 2017). Additionally, the high relative percentage of Cyanobacteria was previously reported to have significant impacts on the variation of ARGs (Guo et al., 2018). Wang et al. (2018) showed that Proteobacteria, Actinobacteria and Cyanobacteria in the environment can carry some ARGs, confirming the high normalized abundance of ARGs and the vital roles of these bacteria in the dissemination of ARGs. Furthermore, we found a significant seasonality in the microbial taxonomic community, and the Procrustes test indicated the asynchronous temporal patterns between microbial OTUs and ARGs compositions (Figure 2). Previous studies have shown that ARGs strongly correlate with microbial communities in antibiotic-rich environments including human gut (Feng et al., 2018) and landfill leachate (Zhao et al., 2018). Recently, Ma et al. (2017) indicated that ARGs and bacterial communities did not show a strong correlation in drinking water, which is similar to the result of our study under low or medium antibiotic pressure. Altogether, the weak correlation between ARGs and bacterial communities in this study may be due to the relatively low antibiotic and heavy metal selection pressure in the natural environment.

### The Co-occurrence Pattern of Bacterial OTUs and ARGs

Previous studies have confirmed that network analysis is a useful and reliable method to decipher the co-occurrence patterns between bacterial taxa and ARG subtypes, and to track potential hosts of ARGs (Barberán et al., 2012; Li et al., 2015; Ma et al., 2017; Feng et al., 2018). The module hubs in the network function as keystone of the module (Faust and Raes, 2012). All module hubs in our co-occurrence network were affiliated to ARGs, implying that ARGs were important in maintaining the structure of the network and that HGT of ARGs was frequent among bacteria in the microbial community. The high frequency of aminoglycoside resistance genes in the network may be due to their high abundance and richness in the community. However, the abundance and richness of tetracycline resistance genes were relatively low in our studied system, but they connected tightly with various bacterial taxa. This suggests that the tetracycline resistance genes were shared among multiple bacterial taxa (Li et al., 2015). An increasing number of studies have reported the widespread distribution of tetracycline resistance genes (Czekalski et al., 2014; Zhou et al., 2017), as this antibiotic has been widely used in China (Hvistendahl, 2012).

In the co-occurrence network, Proteobacteria, Bacteroidetes and Firmicutes were the most prevalent phyla. These taxa were predicted as possible ARG hosts, and/or they originated from a similar source including upstream wastewater and surface runoff, suggesting the potential dissemination risk of ARGs with these taxa. In fact, Proteobacteria include a wide variety of pathogens and occur frequently as opportunistic pathogens, and have been found in soils that contained almost all major ARG types (Forsberg et al., 2014). Moreover, Jang et al. (2018) found that Bacteroidetes can be potential hosts of the *intI-1* gene. In this study, Bacteroidetes were significantly correlated to all major ARG types except sulfonamide resistance genes (Figure 3 and Supplementary Table S2), suggesting the potential transmission of ARGs to human pathogenic bacteria. The Firmicutes were strongly correlated to aminoglycoside resistance genes in sewage sludge (Su et al., 2015), and the ARGs were shown to frequently switch hosts from Firmicutes to other bacterial phyla (Wang et al., 2017). In our study, Firmicutes were not the dominant bacteria but may carry diverse ARGs and further cause ARGs pollution to different environments or ecosystems. Nevertheless, the co-occurrence patterns revealed by this study were based on correlation, so the exact ARG carrying host and transmission pathway of ARGs need to be verified by further study.

### Assembly Mechanisms Driving the Dynamics of ARGs

Our results indicated that the variation in ARGs could be explained by the bacterial community, MGEs and environmental factors. Environmental factors including salinity, total carbon, nitrate nitrogen, total phosphorus and precipitation influenced the temporal variation of ARGs, while precipitation was the strongest factor. A recent study reported that nutrients explained a large part of ARGs variation and that the nutrients combined with other factors could drive the distribution of ARGs (Zhao et al., 2017). The seasonal stormwater runoff induced by rainfall can increase the absolute abundance of ARGs and MGEs in an aquatic environment (Chen et al., 2019) and could influence the ARGs load through increased input from runoff and soil resuspension (Di Cesare et al., 2017). In accordance with previous studies, we found that precipitation displayed a significant influence on the ARG composition and structure, and was positively correlated with the richness and normalized abundance of ARGs, supporting the view that rainfall contributed to the loading of ARGs in the river and reservoir waters in urban region. In addition, rainfall significantly influenced pathogenic bacterial abundance because stormwater was able to significantly contribute to the occurrence and elevated concentration of pathogenic bacteria in subtropical waters (Ahmed et al., 2018). Our results further showed that turbidity was strongly correlated to richness and diversity of bacteria and ARGs. A turbidity increase is normally associated with rainfall-induced contamination, suspended particulate matter and sediment disturbance. This may further drive changes in bacterial community and ARG profiles. It has been demonstrated that turbidity was significantly correlated to bacterial diversity, reiterating the influence of runoff on the bacterial community (Peter and Sommaruga, 2016). Taken together, our results indicated that precipitation had both direct and indirect effects on bacterial taxonomic communities and resistomes, supporting the idea that stormwater with agriculture/urban runoff, with contaminants from the urban region could increase loadings of bacterial ARGs thereby increasing the risk of ARGs dissemination (Garner et al., 2017). In contrast, we did not find any significant relationship between water temperature and the ARGs composition or abundance, corroborating the fact that the ARGs in this subtropical urban reservoir did not have a significant seasonal pattern. This is inconsistent with a previous finding that water temperature was a potential factor driving the dynamics of ARG profiles (Zheng et al., 2018), and this inconsistency may be attributed to a difference in sampling frequency and climate conditions, as the range of temperature was narrower in this subtropical reservoir compared to other studies in temperate zone. In the Zheng et al. (2018) study, water samples were only collected in March, June, September, and December, and their river showed a wider temperature range than our studied reservoir. In our study, the 42.49% of ARGs variance remained unexplained, may partly be due to the environmental factors that were not considered, such as anthropogenic factors and the co-selection of antibiotics and heavy metals (Stepanauskas et al., 2006; Liu et al., 2018; Chen et al., 2019).

The bacterial community has been identified as one of the key drivers that shape the ARG profiles in antibiotic-rich environments (Forsberg et al., 2014; Su et al., 2015). However, our results of pRDA, Procrustes test and Spearman’s relationship all showed that microbial community was weakly correlated with ARG profiles. More interestingly, our results indicated that the neutral process played a more important role in the assembly of microbial taxonomic communities than in ARG profiles, as the neutral model only explained a minor part (7.9%) of the variation in ARG profiles. Therefore, dispersal and stochastic processes appeared to have more influence on bacterial taxonomic communities compared to ARG profiles. This may be one of the reasons why microbial OTUs and ARGs communities exhibited a decoupling change over 1 year. These results indicated that the vertical transmission (mother-to-child transmission) of ARGs in this environment may not play a leading role.

Our study showed that MGEs could partly explain the variation of ARG profiles in the urban reservoir, and the absolute abundance of MGEs showed a significant and positive correlation with ARGs. Further, MGEs that were always abundant in our samples, could potentially enhance the probability of HGT of ARGs. Previous studies reported that environmental contaminants at low concentration have a potential role in the dissemination of ARGs by promoting HGT, such as antibiotic and non-antibiotic pharmaceuticals (Andersson and Hughes, 2014; Wang et al., 2019). In this study, the rainfall combined with possible contaminants and MGEs may largely facilitate the proliferation and spread of ARGs among different bacterial taxa. These results suggest that MGEs played an important role in the temporal variation of ARGs in the urban waterbody, implying the potential risk that the ARGs may be transferred by MGEs from aquatic environments to human pathogens (Martínez et al., 2015).

## Conclusion

Our weekly study revealed the asynchronous succession patterns of bacterial taxonomic communities and ARG profiles, indicating a decoupling dynamic between bacterial taxonomy and function on a temporal scale in a subtropical urban reservoir. The bacterial taxonomic community was significantly correlated with water temperature and exhibited seasonality, while ARG profiles was strongly correlated with precipitation and did not show distinct seasonality. Precipitation had both direct and indirect effects on the temporal patterns of bacterial community and ARG profiles, reflecting the effect of stormwater runoff on temporal dynamics of ARG profiles. The diversity and composition of bacterial taxonomic communities appeared to be more responsive to environmental variables than ARGs, and the composition of ARG profiles appeared to be governed less by stochastic processes. Additionally, MGEs were positively correlated with ARGs and had significant contribution to the variation of ARGs community, suggesting the potential risk of HGT. This study may help to better understand the temporal dynamic of ARG profiles in urban water environments at fine temporal scale, and highlight that further high-frequency and long-term time series research should be undertaken to reveal the specific drivers and deep mechanisms of ARGs assembly and dynamics.

## Data Availability

The raw data for 16S rRNA gene analysis in this study have been deposited in the NCBI SRA database under the BioProject number PRJNA510463 and the accession number SRP173857.

## Author Contributions

JY designed the experiments. PF, FP, and XG carried out the sample collection and determined the environmental parameters. PF, FP, and PX conducted the PCR, high-throughput qPCR, high-throughput sequencing, and bioinformatics. PF and JY wrote the first draft of the manuscript. All authors contributed to and have approved the final manuscript.

## Conflict of Interest Statement

The authors declare that the research was conducted in the absence of any commercial or financial relationships that could be construed as a potential conflict of interest.

## References

[B1] Ahmed W. Zhang Q. Lobos A. Senkbeil J. Sadowsky M. J. Harwood V. J. (2018). Precipitation influences pathogenic bacteria and antibiotic resistance gene abundance in storm drain outfalls in coastal sub-tropical waters. *Environ. Int.* 116 308–318. 10.1016/j.envint.2018.04.005 29754026

[B2] An X. L. Su J. Q. Li B. Ouyang W. Y. Zhao Y. Chen Q. L. (2018). Tracking antibiotic resistome during wastewater treatment using high throughput quantitative PCR. *Environ. Int.* 117 146–153. 10.1016/j.envint.2018.05.011 29751164

[B3] Andersson D. I. Hughes D. (2014). Microbiological effects of sublethal levels of antibiotics. *Nat. Rev. Microbiol.* 12 465–478. 10.1038/nrmicro3270 24861036

[B4] Barberán A. Bates S. T. Casamayor E. O. Fierer N. (2012). Using network analysis to explore co-occurrence patterns in soil microbial communities. *ISME J.* 6 343–351. 10.1038/ismej.2011.119 21900968 PMC3260507

[B5] Berendonk T. U. Manaia C. M. Merlin C. Fatta-Kassinos D. Cytryn E. Walsh F. (2015). Tackling antibiotic resistance: the environmental framework. *Nat. Rev. Microbiol.* 13 310–317. 10.1038/nrmicro3439 25817583

[B6] Börjesson S. Melin S. Matussek A. Lindgren P. E. (2009). A seasonal study of the mecA gene and *Staphylococcus aureus* including methicillin-resistant *S. aureus* in a municipal wastewater treatment plant. *Water Res.* 43 925–932. 10.1016/j.watres.2008.11.036 19084256

[B7] Chen Y. Su J. Q. Zhang J. Li P. Chen H. Zhang B. (2019). High-throughput profiling of antibiotic resistance gene dynamic in a drinking water river-reservoir system. *Water Res.* 149 179–189. 10.1016/j.watres.2018.11.007 30447523

[B8] Clarke K. R. Gorley R. N. (2015). *PRIMER v7: User Manual/Tutorial*. Plymouth: PRIMER-E Ltd.

[B9] Czekalski N. Diez E. G. Bürgmann H. (2014). Wastewater as a point source of antibiotic-resistance genes in the sediment of a freshwater lake. *ISME J.* 8 1381–1390. 10.1038/ismej.2014.8 24599073 PMC4069405

[B10] D’Costa V. M. King C. E. Kalan L. Morar M. Sung W. W. L. Schwarz C. (2011). Antibiotic resistance is ancient. *Nature* 477 457–461. 10.1038/nature10388 21881561

[B11] DeSantis T. Z. Hugenholtz P. Larsen N. Rojas M. Brodie E. L. Keller K. (2006). Greengenes, a chimera-checked 16S rRNA gene database and workbench compatible with ARB. *Appl. Environ. Microbiol.* 72 5069–5072. 10.1128/AEM.03006-05 16820507 PMC1489311

[B12] Di Cesare A. Eckert E. M. Rogora M. Corno G. (2017). Rainfall increases the abundance of antibiotic resistance genes within a riverine microbial community. *Environ. Pollut.* 226 473–478. 10.1016/j.envpol.2017.04.036 28438356

[B13] Edgar R. C. (2010). Search and clustering orders of magnitude faster than BLAST. *Bioinformatics* 26 2460–2461. 10.1093/bioinformatics/btq461 20709691

[B14] Faust K. Raes J. (2012). Microbial interactions: from networks to models. *Nat. Rev. Microbiol.* 10 538–550. 10.1038/nrmicro2832 22796884

[B15] Feng J. Li B. Jiang X. Yang Y. Wells G. F. Zhang T. (2018). Antibiotic resistome in a large-scale healthy human gut microbiota deciphered by metagenomic and network analyses. *Environ. Microbiol.* 20 355–368. 10.1111/1462-2920.14009 29194931

[B16] Forsberg K. J. Patel S. Gibson M. K. Lauber C. L. Knight R. Fierer N. (2014). Bacterial phylogeny structures soil resistomes across habitats. *Nature* 509 612–616. 10.1038/nature13377 24847883 PMC4079543

[B17] Garner E. Benitez R. von Wagoner E. Sawyer R. Schaberg E. Hession W. C. (2017). Stormwater loadings of antibiotic resistance genes in an urban stream. *Water Res.* 123 144–152. 10.1016/j.watres.2017.06.046 28662396

[B18] Guo Y. Y. Liu M. Liu L. M. Liu X. Chen H. H. Yang J. (2018). The antibiotic resistome of free-living and particle-attached bacteria under a reservoir cyanobacterial bloom. *Environ. Int.* 117 107–115. 10.1016/j.envint.2018.04.045 29734061

[B19] Hubbell S. P. (2001). *The Unified Neutral Theory of Biodiversity and Biogeography (MPB-32)*. Princeton, NJ: Princeton University Press.

[B20] Hvistendahl M. (2012). China takes aim at rampant antibiotic resistance. *Science* 336 795–795. 10.1126/science.336.6083.795 22605727

[B21] Jang H. M. Kim Y. B. Choi S. Lee Y. Shin S. G. Unno T. (2018). Prevalence of antibiotic resistance genes from effluent of coastal aquaculture, South Korea. *Environ. Pollut.* 233 1049–1057. 10.1016/j.envpol.2017.10.006 29031406

[B22] Jia S. Shi P. Hu Q. Li B. Zhang T. Zhang X. X. (2015). Bacterial community shift drives antibiotic resistance promotion during drinking water chlorination. *Environ. Sci. Technol.* 49 12271–12279. 10.1021/acs.est.5b03521 26397118

[B23] Jia S. Zhang X. X. Miao Y. Zhao Y. Ye L. Li B. (2017). Fate of antibiotic resistance genes and their associations with bacterial community in livestock breeding wastewater and its receiving river water. *Water Res.* 124 259–268. 10.1016/j.watres.2017.07.061 28763642

[B24] Kruse H. Sørum H. (1994). Transfer of multiple drug resistance plasmids between bacteria of diverse origins in natural microenvironments. *Appl. Environ. Microbiol.* 60 4015–4021. 11865872 10.1128/aem.60.11.4015-4021.1994 PMC201930

[B25] Laxminarayan R. (2014). Antibiotic effectiveness: balancing conservation against innovation. *Science* 345 1299–1301. 10.1126/science.1254163 25214620

[B26] Li B. Yang Y. Ma L. Ju F. Guo F. Tiedje J. M. (2015). Metagenomic and network analysis reveal wide distribution and co-occurrence of environmental antibiotic resistance genes. *ISME J.* 9 2490–2502. 10.1038/ismej.2015.59 25918831 PMC4611512

[B27] Lindh M. V. Sjöstedt J. Andersson A. F. Baltar F. Hugerth L. W. Lundin D. (2015). Disentangling seasonal bacterioplankton population dynamics by high-frequency sampling. *Environ. Microbiol.* 17 2459–2476. 10.1111/1462-2920.12720 25403576

[B28] Liu L. M. Su J. Q. Guo Y. Y. Wilkinson D. M. Liu Z. W. Zhu Y. G. (2018). Large-scale biogeographical patterns of bacterial antibiotic resistome in the waterbodies of China. *Environ. Int.* 117 292–299. 10.1016/j.envint.2018.05.023 29891393

[B29] Liu L. M. Yang J. Yu X. Q. Chen G. J. Yu Z. (2013). Patterns in the composition of microbial communities from a subtropical river: effects of environmental, spatial and temporal factors. *PLoS One* 8:e81232 10.1371/journal.pone.0081232 PMC3828266 24244735

[B30] Logares R. Lindström E. S. Langenheder S. Logue J. B. Paterson H. Laybourn-Parry J. (2013). Biogeography of bacterial communities exposed to progressive long-term environmental change. *ISME J.* 7 937–948. 10.1038/ismej.2012.168 23254515 PMC3635229

[B31] Louca S. Parfrey L. W. Doebeli M. (2016). Decoupling function and taxonomy in the global ocean microbiome. *Science* 353 1272–1277. 10.1126/science.aaf4507 27634532

[B32] Ma L. Li B. Jiang X. T. Wang Y. L. Xia Y. Li A. D. (2017). Catalogue of antibiotic resistome and host-tracking in drinking water deciphered by a large scale survey. *Microbiome* 5:154 10.1186/s40168-017-0369-0 PMC5704573 29179769

[B33] Marti E. Variatza E. Balcazar J. L. (2014). The role of aquatic ecosystems as reservoirs of antibiotic resistance. *Trends Microbiol.* 22 36–41. 10.1016/j.tim.2013.11.001 24289955

[B34] Martínez J. L. Coque T. M. Baquero F. (2015). What is a resistance gene? Ranking risk in resistomes. *Nat. Rev. Microbiol.* 13 116–123. 10.1038/nrmicro3399 25534811

[B35] Ofiţeru I. D. Lunn M. Curtis T. P. Wells G. F. Criddle C. S. Francis C. A. (2010). Combined niche and neutral effects in a microbial wastewater treatment community. *Proc. Natl. Acad. Sci. U.S.A.* 107 15345–15350. 10.1073/pnas.1000604107 20705897 PMC2932620

[B36] Ouyang W. Y. Huang F. Y. Zhao Y. Li H. Su J. Q. (2015). Increased levels of antibiotic resistance in urban stream of Jiulongjiang River, China. *Appl. Microbiol. Biotechnol.* 99 5697–5707. 10.1007/s00253-015-6416-5 25661810

[B37] Peter H. Sommaruga R. (2016). Shifts in diversity and function of lake bacterial communities upon glacier retreat. *ISME J.* 10 1545–1554. 10.1038/ismej.2015.245 26771929 PMC4852812

[B38] Pruden A. Pei R. Storteboom H. Carlson K. H. (2006). Antibiotic resistance genes as emerging contaminants: studies in northern Colorado. *Environ. Sci. Technol.* 40 7445–7450. 10.1021/es060413l 17181002

[B39] Schloss P. D. Westcott S. L. Ryabin T. Hall J. R. Hartmann M. Hollister E. B. (2009). Introducing mothur: open-source, platform-independent, community-supported software for describing and comparing microbial communities. *Appl. Environ. Microbiol.* 75 7537–7541. 10.1128/AEM.01541-09 19801464 PMC2786419

[B40] Sloan W. T. Lunn M. Woodcock S. Head L. M. Nee S. Curtis T. P. (2006). Quantifying the roles of immigration and chance in shaping prokaryote community structure. *Environ. Microbiol.* 8 732–740. 10.1111/j.1462-2920.2005.00956.x 16584484

[B41] Stepanauskas R. Glenn T. C. Jagoe C. H. Tuckfield R. C. Lindell A. H. King C. J. (2006). Coselection for microbial resistance to metals and antibiotics in freshwater microcosms. *Environ. Microbiol.* 8 1510–1514. 10.1111/j.1462-2920.2006.01091.x 16913911

[B42] Su J. Q. Wei B. Ouyang W. Y. Huang F. Y. Zhao Y. Xu H. J. (2015). Antibiotic resistome and its association with bacterial communities during sewage sludge composting. *Environ. Sci. Technol.* 49 7356–7363. 10.1021/acs.est.5b01012 26018772

[B43] Treusch A. H. Vergin K. L. Finlay L. A. Donatz M. G. Burton R. M. Carlson C. A. (2009). Seasonality and vertical structure of microbial communities in an ocean gyre. *ISME J.* 3 1148–1163. 10.1038/ismej.2009.60 19494846

[B44] Wang C. Dong D. Strong P. J. Zhu W. Ma Z. Qin Y. (2017). Microbial phylogeny determines transcriptional response of resistome to dynamic composting processes. *Microbiome* 5 1–15. 10.1186/s40168-017-0324-0 28814344 PMC5559795

[B45] Wang J. H. Lu J. Zhang Y. X. Wu J. Luo Y. Liu H. (2018). Metagenomic analysis of antibiotic resistance genes in coastal industrial mariculture systems. *Bioresour. Technol.* 253 235–243. 10.1016/j.biortech.2018.01.035 29353751

[B46] Wang Y. Lu J. Mao L. Li J. Yuan Z. Bond P. L. (2019). Antiepileptic drug carbamazepine promotes horizontal transfer of plasmid-borne multi-antibiotic resistance genes within and across bacterial genera. *ISME J.* 13 509–522. 10.1038/s41396-018-0275-x 30291330 PMC6331567

[B47] World Health Organization [WHO] (2014). *Antimicrobial Resistance: Global Report on Surveillance 2014*. Geneva: World Health Organization.

[B48] Xu L. Ouyang W. Qian Y. Su C. Su J. Chen H. (2016). High-throughput profiling of antibiotic resistance genes in drinking water treatment plants and distribution systems. *Environ. Pollut.* 213 119–126. 10.1016/j.envpol.2016.02.013 26890482

[B49] Yang J. Yu X. Q. Liu L. M. Zhang W. J. Guo P. Y. (2012). Algae community and trophic state of subtropical reservoirs in southeast Fujian, China. *Environ. Sci. Pollut. Res.* 19 1432–1442. 10.1007/s11356-011-0683-1 22743992

[B50] Yang Y. Li B. Ju F. Zhang T. (2013). Exploring variation of antibiotic resistance genes in activated sludge over a four-year period through a metagenomic approach. *Environ. Sci. Technol.* 47 10197–10205. 10.1021/es4017365 23919449

[B51] Yang Y. Song W. Lin H. Wang W. Du L. Xing W. (2018). Antibiotics and antibiotic resistance genes in global lakes: a review and meta-analysis. *Environ. Int.* 116 60–73. 10.1016/j.envint.2018.04.011 29653401

[B52] Zhang H. Feng J. Chen S. Zhao Z. Li B. Wang Y. (2019). Geographical patterns of *nirS* gene abundance and *nirS*-type denitrifying bacterial community associated with activated sludge from different wastewater treatment plants. *Microb. Ecol.* 77 304–316. 10.1007/s00248-018-1236-7 30046860

[B53] Zhao R. Feng J. Yin X. Liu J. Fu W. Berendonk T. U. (2018). Antibiotic resistome in landfill leachate from different cities of China deciphered by metagenomic analysis. *Water Res.* 134 126–139. 10.1016/j.watres.2018.01.063 29407646

[B54] Zhao Z. Wang J. Han Y. Chen J. Liu G. Lu H. (2017). Nutrients, heavy metals and microbial communities co-driven distribution of antibiotic resistance genes in adjacent environment of mariculture. *Environ. Pollut.* 220 909–918. 10.1016/j.envpol.2016.10.075 27814984

[B55] Zheng J. Zhou Z. Wei Y. Chen T. Feng W. Chen H. (2018). High-throughput profiling of seasonal variations of antibiotic resistance gene transport in a peri-urban river. *Environ. Int.* 114 87–94. 10.1016/j.envint.2018.02.039 29499451

[B56] Zhou Y. Niu L. Zhu S. Lu H. Liu W. (2017). Occurrence, abundance, and distribution of sulfonamide and tetracycline resistance genes in agricultural soils across China. *Sci. Total Environ.* 599 1977–1983. 10.1016/j.scitotenv.2017.05.152 28558428

[B57] Zhu Y. G. Zhao Y. Li B. Huang C. L. Zhang S. Y. Yu S. (2017). Continental-scale pollution of estuaries with antibiotic resistance genes. *Nat. Microbiol.* 2:16270 10.1038/nmicrobiol.2016.270 28134918

