# Supplementary material for: Decoupling the Dynamics of Bacterial Taxonomy and Antibiotic Resistance Function in a Subtropical Urban Reservoir as Revealed by High-Frequency Sampling
Source: Front Microbiol. 2019 Jul 2;10:1448. doi: 10.3389/fmicb.2019.01448 (PMC6614491; doi:10.3389/fmicb.2019.01448)
Supplement: Supplementary file 1 [file Table_1.doc]

**Journal: Frontiers in Microbiology**

*Supplementary information of the article:*

**Decoupling the dynamics of bacterial taxonomy and antibiotic resistance function in a subtropical urban reservoir as revealed by high-frequency sampling**

Peiju Fang^1, 2^, Feng Peng^1, 2^, Xiaofei Gao^1, 2^, Peng Xiao^1^, Jun Yang^1^, *

^1^ *Aquatic Ecohealth Group, Key Laboratory of Urban Environment and Health, Institute of Urban Environment, Chinese Academy of Sciences, Xiamen 361021, China,*

^2^ *University of Chinese Academy of Sciences, Beijing 100049, China*

***Correspondence**

*E-mail address:* [jyang@iue.ac.cn](mailto:jyang@iue.ac.cn) (Jun Yang); Tel. / Fax: +86 592-6190775.

**This supplementary information contains:**

- 15 Pages
- 8 Figures
- 6 Tables

**
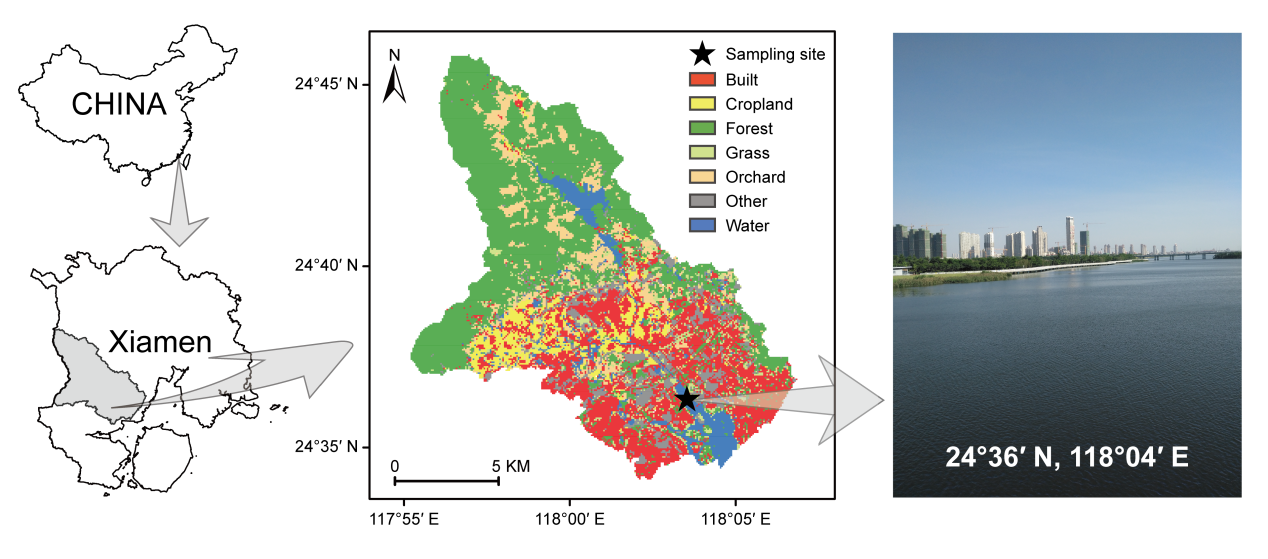
**

**FIGURE S1 |** Location of the sampling site in Xinglinwan Reservoir, Xiamen city, southeast China.


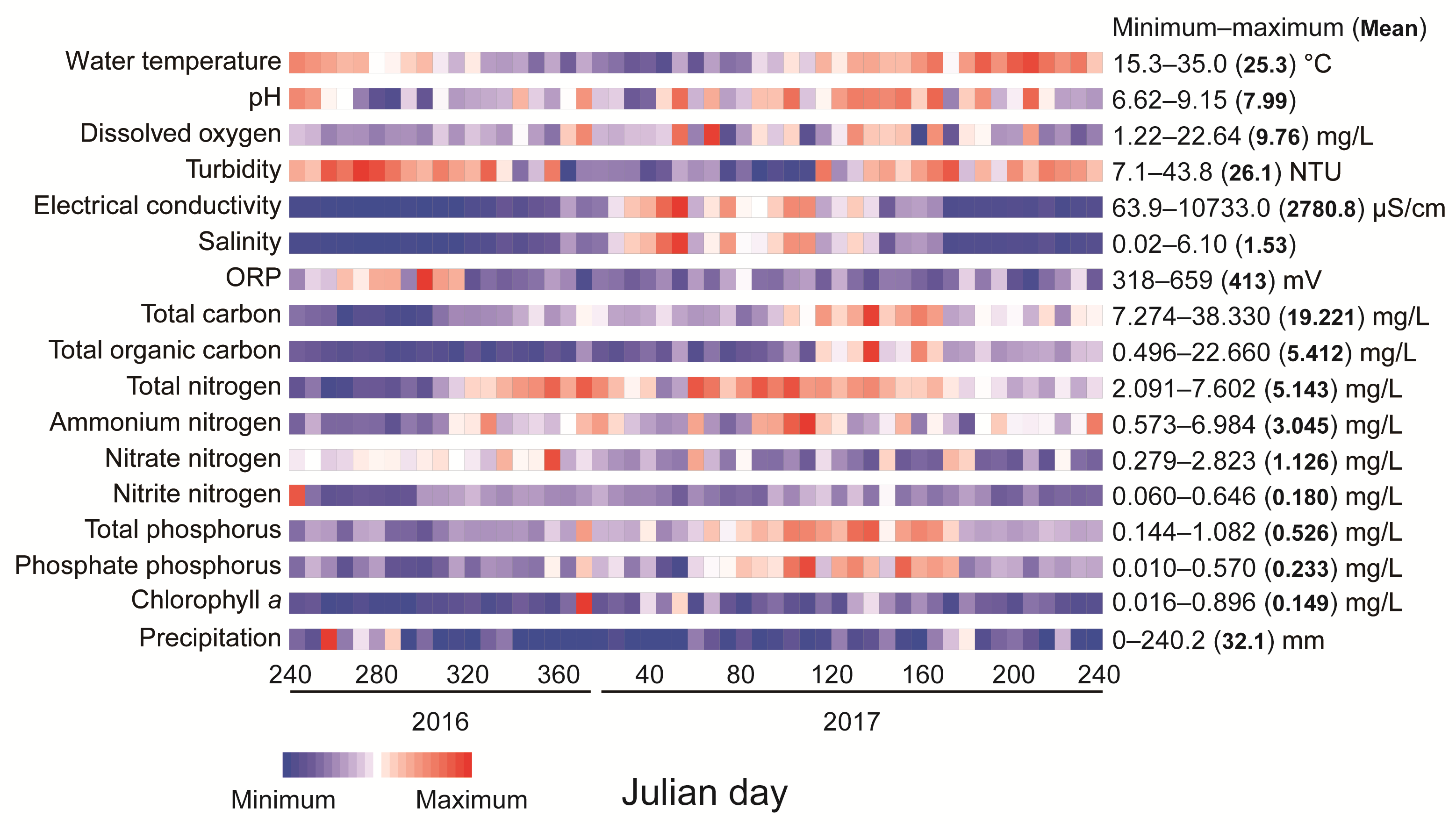


**FIGURE S2 |** Heatmap showing the range of 17 environmental factors of Xinglinwan Reservoir. ORP, oxidation reduction potential.


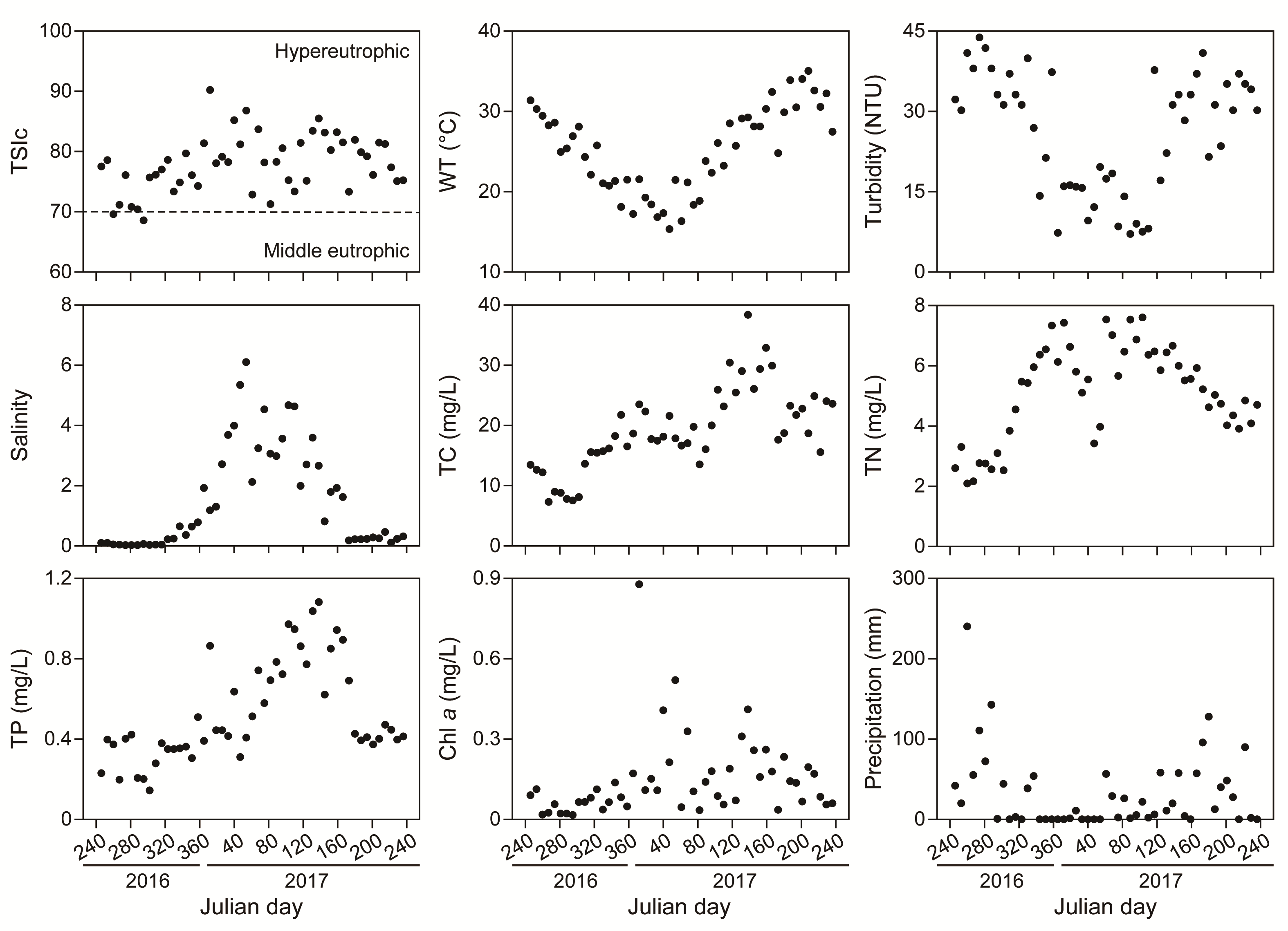


**FIGURE S3 |** Temporal dynamics of major environmental factors in Xinglinwan Reservoir. TSIc, comprehensive trophic state index; WT, water temperature; TC, total carbon; TN, total nitrogen; TP, total phosphorus; Chl *a*, chlorophyll *a*; precipitation refers to the cumulative rainfall during the last week before the sampling day.


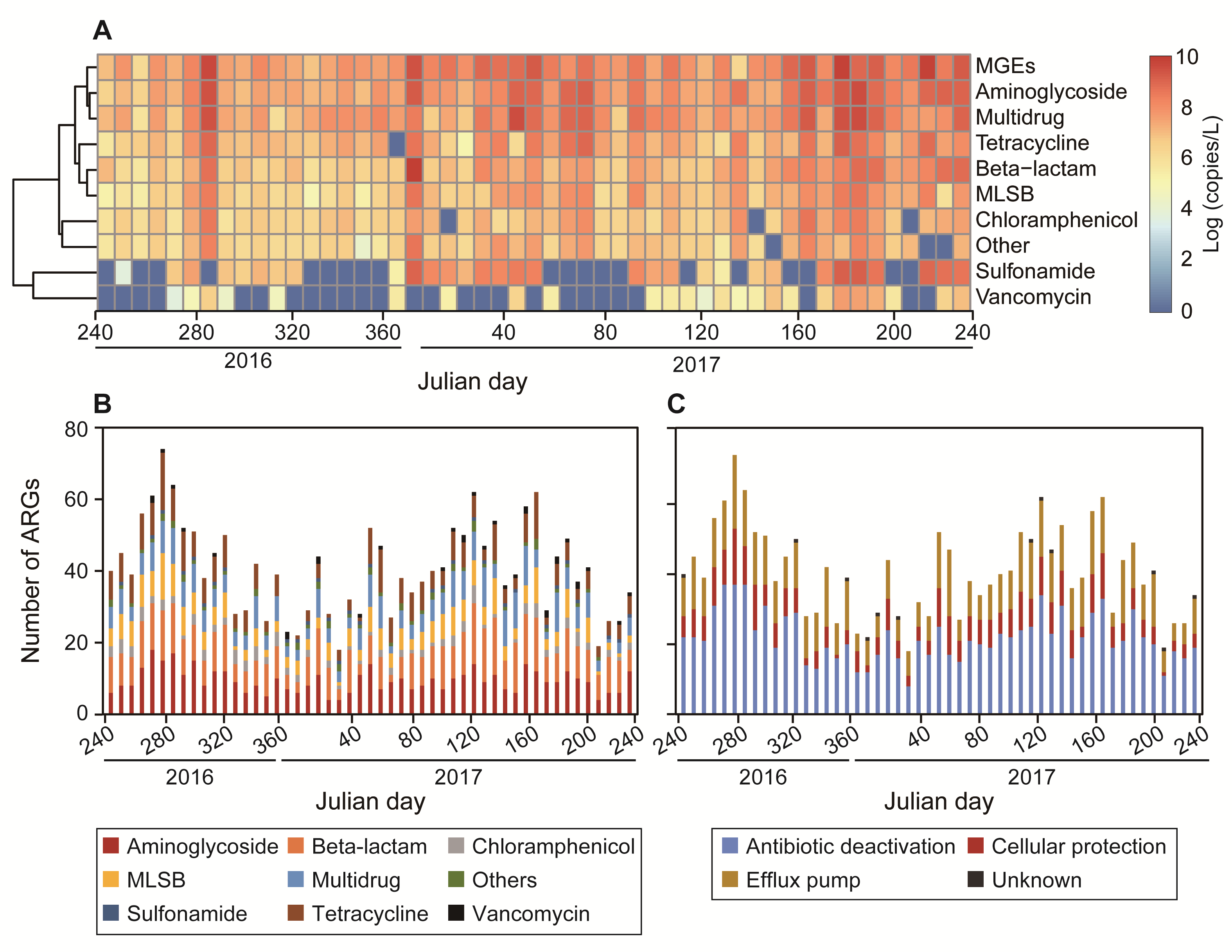


**FIGURE S4 |** Composition of antibiotic resistance genes in Xinglinwan Reservoir. (**A**) Profiling of ARG and MGE types based on the absolute abundance [log (copies/L)], ranging from low (blue) to high (red) values. The number of detected resistance genes which were classified based on (**B**) the antibiotic they confer resistance to and (**C**) the mechanism of resistance.


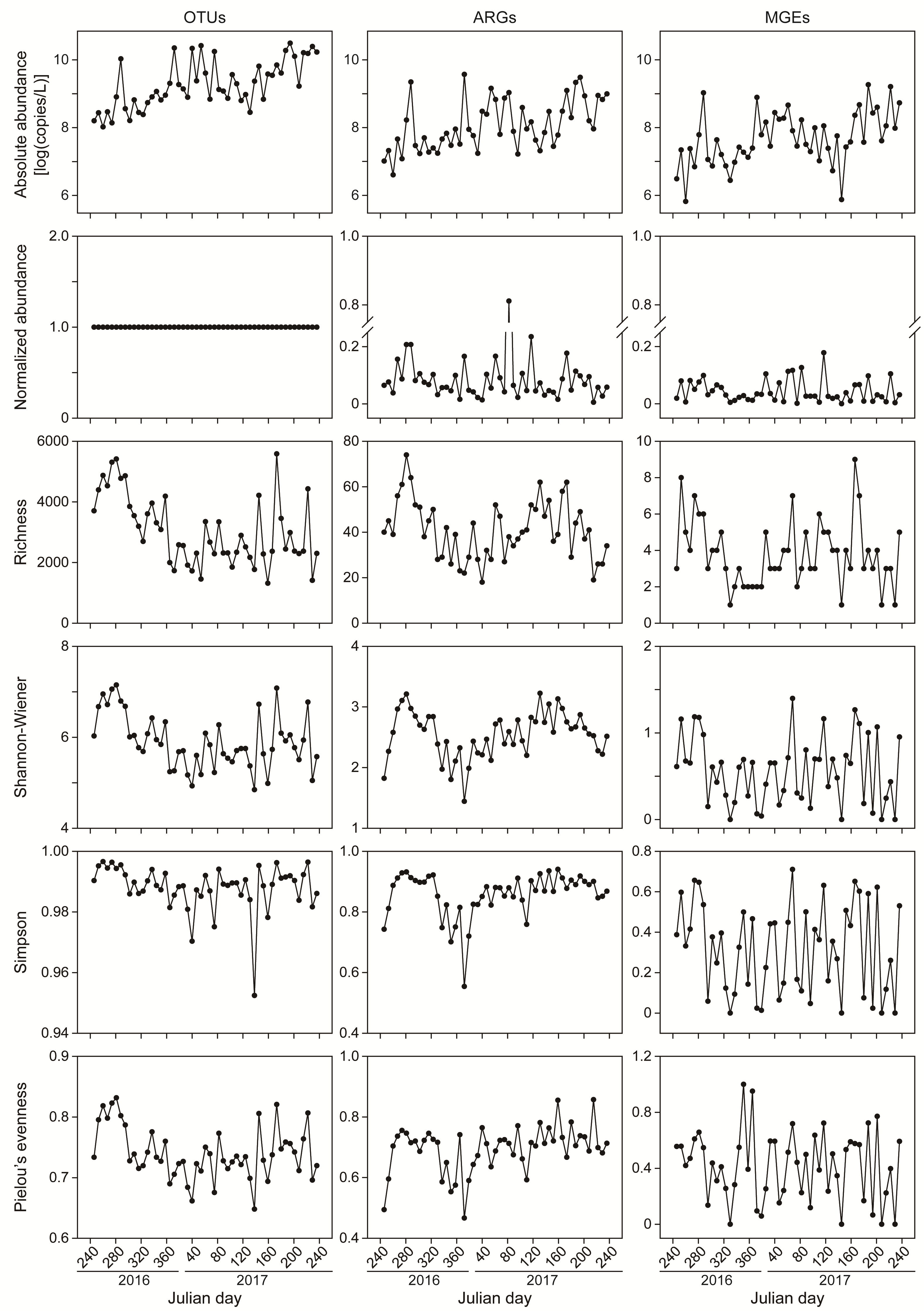


**FIGURE S5 |** The abundance, richness and diversity of bacterioplankton OTUs, ARGs and MGEs from Xinglinwan Reservoir.

**
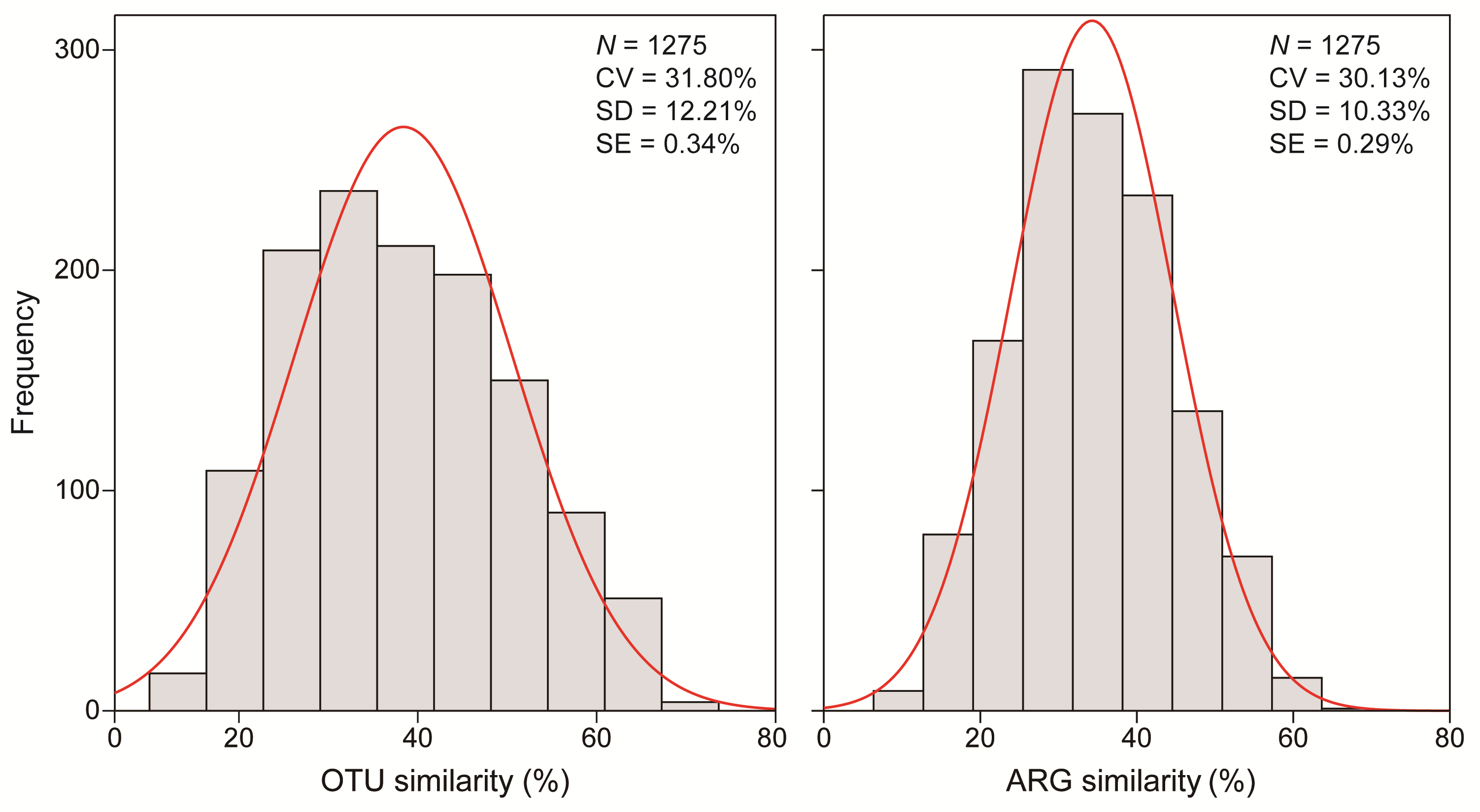
FIGURE S6 |** The frequency distributions of Bray-Curtis similarities of bacterial community (**left**) and ARG profiles (**right**) from Xinglinwan Reservoir. CV means variation coefficient of community similarity, SD is standard deviation, and SE is standard error.


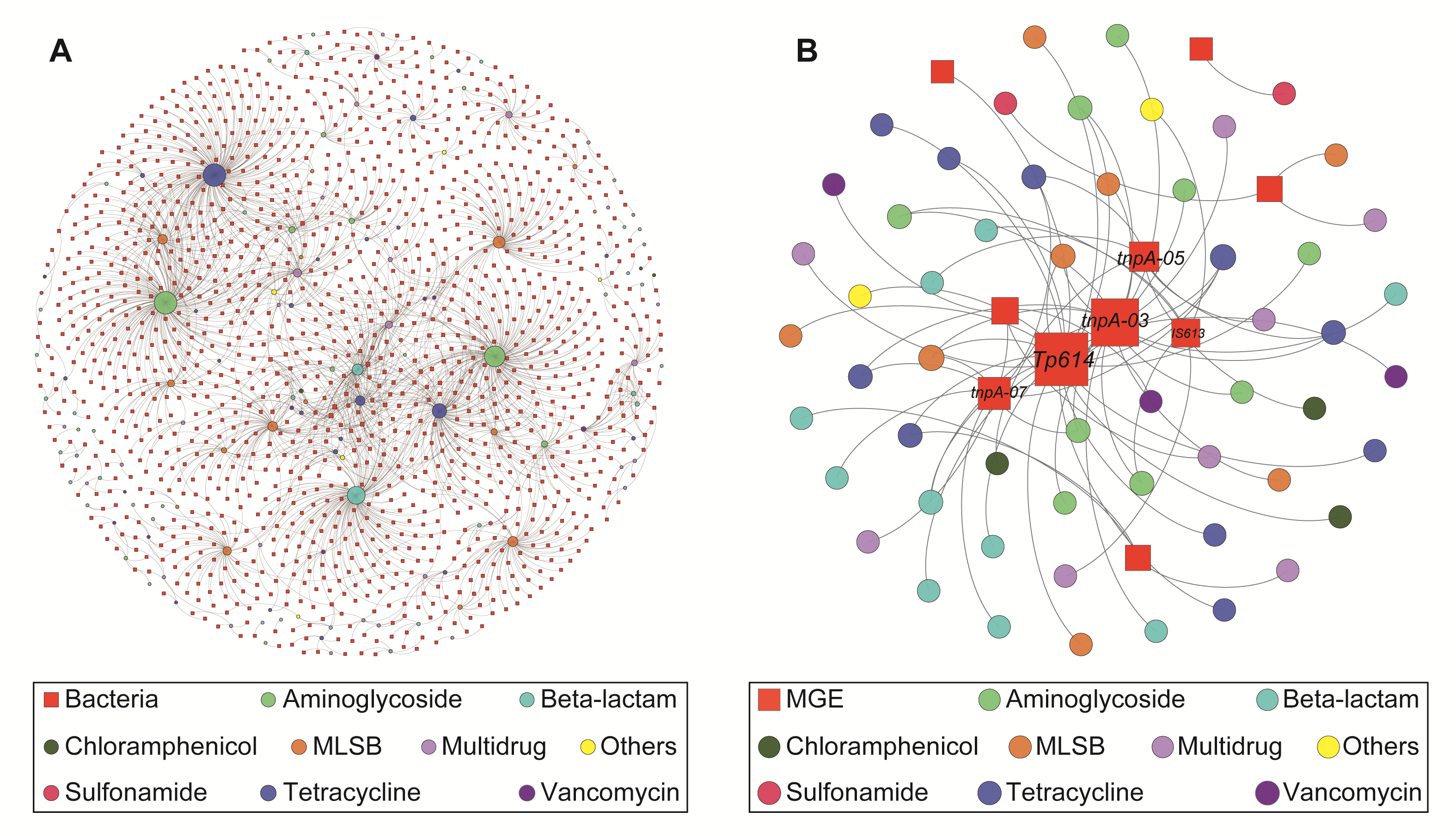


**FIGURE S7 |** Co-occurrence network showing significant correlations between (**A**) bacterial OTUs and ARGs, (**B**) MGEs and ARGs. The color of nodes is assigned based on ARG types, and the size of each node corresponds to number of connections.


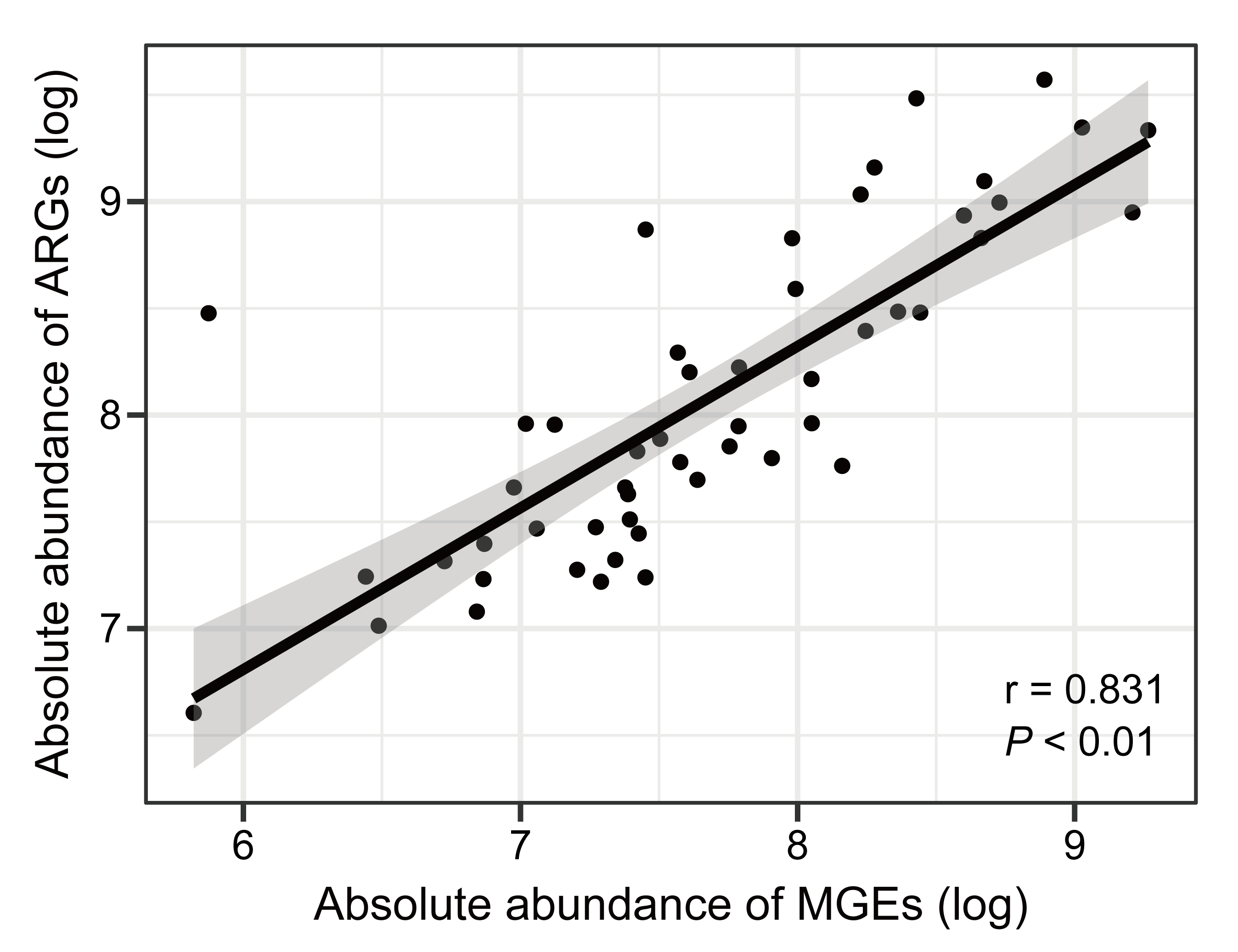


**FIGURE S8 |** Relationship between absolute abundance of MGEs and absolute abundance of ARGs. The coefficient r value and significance *P*-value are derived from Spearman’s correlation (*n* = 51).

**Table S1 | Major contributors of temporal compositional change for bacterial taxonomy and ARG profiles assessed by the similarity percentage (SIMPER) analysis.**

| Phylum | Contribution (%) | Type | Contribution (%) |
| --- | --- | --- | --- |
| Actinobacteria | 16.31 | Aminoglycoside | 22.41 |
| Bacteroidetes | 12.19 | Beta-lactam | 23.41 |
| Cyanobacteria | 10.16 | Chloramphenicol | 5.13 |
| Firmicutes | 4.74 | MLSB | 11.88 |
| Other | 11.2 | Multidrug | 17.04 |
| Planctomycetes | 4.12 | Sulfonamide | 1.65 |
| Proteobacteria | 31.86 | Tetracycline | 14.12 |
| Unclassified | 5.13 | Vancomycin | 2.15 |
| Verrucomicrobia | 4.29 | Other | 2.21 |

**Table S2 |** Mantel tests showing Spearman’s relationship between bacterial taxonomy and function at OTU and ARG subtype levels.

| Taxa | Aminoglycoside | Beta-lactam | Chloramphenicol | MLSB | Multidrug | Others | Sulfonamide | Tetracycline | Vancomycin | MGEs |
| --- | --- | --- | --- | --- | --- | --- | --- | --- | --- | --- |
| Actinobacteria | **0.186**** | **0.117**** | 0.051 | **0.100*** | **0.148**** | –0.008 | –0.003 | **0.150**** | **0.239**** | 0.005 |
| Bacteroidetes | **0.251**** | **0.307**** | **0.112*** | **0.214**** | **0.210**** | 0.090 | 0.001 | **0.341**** | 0.101 | 0.073 |
| Chlorobi | **0.139**** | **0.173**** | –0.008 | –0.031 | –0.003 | –0.052 | 0.001 | **0.198**** | 0.076 | 0.003 |
| Chloroflexi | **0.193**** | **0.238**** | **0.098*** | **0.157**** | **0.132*** | 0.072 | –0.076 | **0.221**** | **0.195**** | **0.091*** |
| Cyanobacteria | **0.179**** | **0.188**** | **0.137*** | 0.102 | **0.128*** | 0.142 | –0.048 | **0.221**** | **0.228**** | 0.037 |
| Firmicutes | 0.070 | **0.157*** | **0.163*** | **0.156*** | **0.161*** | **0.199*** | –0.113 | 0.076 | **0.133*** | **0.115*** |
| Planctomycetes | **0.145**** | **0.174**** | **0.075*** | **0.102*** | **0.164**** | 0.011 | –0.029 | **0.123*** | **0.134*** | 0.052 |
| Proteobacteria | **0.219**** | **0.292**** | **0.165**** | **0.225**** | **0.227**** | 0.090 | –0.132 | **0.324**** | **0.219**** | 0.078 |
| TM7 | **0.217**** | **0.140**** | **0.101*** | 0.047 | 0.091 | 0.009 | –0.048 | **0.123*** | **0.190**** | **0.092*** |
| Verrucomicrobia | **0.197**** | 0.076 | **0.107*** | **0.234**** | 0.105 | –0.062 | –0.100 | 0.100 | **0.198**** | 0.003 |

The OTU was defined based on 97% sequence similarity level.

MLSB, Macrolide-Lincosamide-Streptogramin B resistance.

Significant correlations (*P* < 0.05) are shown in bold.

* *P* < 0.05, ** *P* < 0.01.

**Table S3 | Mantel test showing the correlations between environmental factors, ARGs and MGEs.**

| Environment variables | Aminoglycoside | Beta-lactam | Chloramphenicol | MLSB | Multidrug | Others | Sulfonamide | Tetracycline | Vancomycin | MGEs |
| --- | --- | --- | --- | --- | --- | --- | --- | --- | --- | --- |
| Environment | **0.131**** | **0.097*** | 0.029 | –0.001 | 0.017 | 0 | –0.038 | **0.198**** | **0.151*** | –0.061 |
| Water temperature | **0.141**** | **0.170**** | –0.002 | 0.099 | 0.072 | 0.029 | 0.007 | **0.138*** | 0.024 | 0.049 |
| pH | 0.024 | –0.009 | –0.043 | –0.080 | –0.039 | 0.016 | 0.037 | **0.139*** | 0.118 | –0.048 |
| Dissolved oxygen | 0.043 | **0.154*** | **0.153*** | 0.058 | –0.036 | 0.143 | –0.041 | 0.148 | 0.042 | 0.115 |
| Turbidity | **0.178**** | 0.062 | 0.058 | –0.024 | –0.007 | –0.002 | 0.071 | 0.005 | –0.062 | 0.047 |
| Electrical conductivity | **0.105*** | 0.010 | –0.005 | –0.028 | 0.042 | –0.040 | –0.054 | 0.022 | **0.127*** | –0.094 |
| Salinity | **0.156**** | 0.072 | 0.058 | –0.013 | 0.052 | 0.027 | –0.058 | **0.122*** | 0.045 | –0.014 |
| ORP | –0.117 | 0.006 | –0.129 | –0.020 | –0.040 | –0.128 | –0.052 | –0.023 | 0.058 | –0.095 |
| Total carbon | –0.033 | –0.075 | –0.004 | –0.117 | –0.007 | 0.058 | 0.113 | 0.003 | 0.122 | –0.123 |
| Total organic carbon | –0.058 | 0.040 | 0.025 | 0.038 | 0.082 | **0.146*** | **0.156*** | 0.004 | 0.049 | –0.002 |
| Total nitrogen | 0.080 | –0.022 | 0.012 | –0.041 | –0.061 | –0.072 | 0.015 | –0.049 | **0.175**** | –0.093 |
| Ammonium nitrogen | 0.006 | 0.021 | –0.006 | –0.081 | –0.091 | 0.005 | –0.144 | –0.009 | 0.110 | –0.026 |
| Nitrate nitrogen | –0.004 | 0.009 | 0.086 | **0.131*** | 0.079 | **0.119*** | 0.004 | 0.068 | 0.007 | 0.068 |
| Nitrite nitrogen | 0.085 | –0.044 | –0.031 | –0.081 | –0.036 | –0.018 | –0.124 | –0.075 | **0.155*** | 0.060 |
| Total phosphorus | 0 | –0.082 | 0.053 | –0.135 | –0.013 | **0.204*** | 0.055 | –0.002 | 0.089 | –0.049 |
| Phosphate phosphorus | –0.019 | –0.077 | **0.113*** | –0.175 | –0.044 | 0.140 | –0.053 | 0.093 | 0.032 | –0.026 |
| Chlorophyll *a* | 0.029 | 0.090 | 0.077 | 0.040 | –0.001 | 0.065 | –0.030 | **0.275**** | 0.056 | –0.027 |
| Precipitation | **0.131**** | **0.110**** | 0.023 | **0.086*** | 0.026 | –0.031 | –0.052 | **0.142**** | **0.122*** | 0.004 |
| MGEs | **0.125*** | **0.114*** | **0.117*** | 0.011 | **0.238**** | –0.014 | 0.023 | 0.025 | 0.051 | **1.000**** |

Environment means the total of following 17 environment variables. ORP, oxidation reduction potential. MLSB, Macrolide-Lincosamide-Streptogramin B resistance; MGEs, mobile genetic elements. Significant correlations (*P* < 0.05) are shown in bold. * *P* < 0.05, ** *P* < 0.01.

**Table S4 |** Spearman’s correlation between bacterial OTUs, ARGs and mobile genetic elements (MGEs).

|  | OTU richness | 16S absolute abundance | A richness | A absolute abundance | A normalized abundance | M absolute abundance | M normalized abundance |
| --- | --- | --- | --- | --- | --- | --- | --- |
| OTU richness | – |  |  |  |  |  |  |
| 16S absolute abundance | **–0.444**** | – |  |  |  |  |  |
| A richness | **0.484**** | **–0.349*** | – |  |  |  |  |
| A absolute abundance | –0.193 | **0.879**** | –0.094 | – |  |  |  |
| A normalized abundance | **0.428**** | –0.095 | **0.579**** | **0.343*** | – |  |  |
| M absolute abundance | –0.273 | **0.756**** | –0.136 | **0.831**** | **0.282*** | – |  |
| M normalized abundance | **0.309*** | –0.206 | **0.396**** | 0.094 | **0.638**** | **0.418**** | – |

The OTU was defined based on 97% sequence similarity level.

16S, 16S rRNA gene; A, ARGs; M, MGEs.

Significant correlations (*P* < 0.05) are shown in bold.

* *P* < 0.05, ** *P* < 0.01.

**Table S5 |** Spearman’s correlation between ARG types and ten MGE marker genes based on **normalized** abundance.

|  | Aminoglycoside | Beta-lactam | Chloramphenicol | MLSB | Multidrug | Others | Sulfonamide | Tetracycline | Vancomycin |
| --- | --- | --- | --- | --- | --- | --- | --- | --- | --- |
| *cIntI-1* | –0.056 | –0.149 | 0.199 | –0.136 | 0.144 | 0.115 | –0.151 | 0.006 | –0.189 |
| *intI-1* | 0.086 | 0.128 | 0.183 | –0.009 | **0**.349* | –0.165 | –0.103 | 0.084 | **0**.286* |
| *IS613* | 0.257 | 0.210 | 0.151 | 0.219 | 0.225 | **0**.431** | –0.055 | 0.190 | –0.256 |
| *tnpA-01* | 0.173 | –0.043 | 0.093 | 0.086 | 0.108 | 0.162 | –0.177 | 0.054 | **0**.281* |
| *tnpA-02* | –0.104 | 0.191 | –0.110 | –0.104 | 0.036 | –0.089 | –0.064 | –0.235 | **0**.342* |
| *tnpA-03* | **0.**461** | 0.255 | **0**.444** | **0**.433** | **0**.432** | **0**.449** | –0.032 | **0**.337* | 0.025 |
| *tnpA-04* | **0**.480** | **0**.302* | 0.277 | **0**.480** | 0.195 | **0**.308* | **0**.412** | **0**.339* | 0.083 |
| *tnpA-05* | **0**.405** | 0.150 | **0**.330* | **0**.345* | **0**.355* | **0**.385** | 0.264 | **0**.470** | 0.061 |
| *tnpA-07* | **0**.489** | 0.229 | **0**.477** | **0**.420** | **0**.296* | **0**.454** | 0.037 | **0**.515** | –0.029 |
| *Tp614* | **0**.368** | 0.203 | **0**.392** | 0.248 | **0**.311* | 0.257 | –0.067 | 0.188 | 0.038 |

The data used were **normalized** abundance (ARGs and MGEs absolute abundance/16S rRNA absolute abundance).

* *P* < 0.05, ** *P* < 0.01.

**Table S6 |** Spearman’s correlation between ARG types and ten MGE marker genes based on **absolute** abundance.

|  | Aminoglycoside | Beta-lactam | Chloramphenicol | MLSB | Multidrug | Others | Sulfonamide | Tetracycline | Vancomycin |
| --- | --- | --- | --- | --- | --- | --- | --- | --- | --- |
| *cIntI-1* | –0.163 | –0.197 | 0.015 | –0.176 | –0.039 | –0.006 | –0.147 | –0.100 | –0.184 |
| *intI-1* | 0.039 | 0.140 | 0.106 | 0.072 | 0.256 | **–0.154** | –0.093 | 0.094 | 0.254 |
| *IS613* | **0.289*** | 0.252 | 0.203 | **0.278*** | **0.377**** | **0.503**** | 0.062 | 0.216 | –0.143 |
| *tnpA-01* | 0.130 | 0.053 | –0.060 | 0.135 | 0.103 | 0.175 | –0.116 | –0.078 | **0.360**** |
| *tnpA-02* | 0.129 | 0.242 | 0.125 | 0.142 | 0.260 | 0.061 | 0.089 | 0.053 | **0.376**** |
| *tnpA-03* | 0.097 | 0.034 | 0.176 | 0.166 | 0.181 | 0.274 | –0.234 | 0.091 | –0.056 |
| *tnpA-04* | **0.311*** | **0.325*** | **0.303*** | 0.248 | 0.196 | 0.055 | **0.453**** | 0.272 | 0.219 |
| *tnpA-05* | **0.512**** | **0.435**** | **0.406**** | **0.481**** | **0.501**** | **0.418**** | **0.334*** | **0.355*** | 0.266 |
| *tnpA-07* | **0.335*** | 0.184 | **0.299*** | **0.324*** | 0.193 | 0.193 | –0.056 | 0.244 | 0.080 |
| *Tp614* | 0.009 | –0.033 | 0.147 | 0.022 | 0.060 | 0.078 | –0.178 | –0.039 | 0.010 |

The data used were **absolute** abundance (copy number per litre water).

* *P* < 0.05, ** *P* < 0.01.
